# Feeding by *Tropilaelaps mercedesae* on pre- and post-capped brood increases damage to *Apis mellifera* colonies

**DOI:** 10.1038/s41598-019-49662-4

**Published:** 2019-09-10

**Authors:** Patcharin Phokasem, Lilia I. de Guzman, Kitiphong Khongphinitbunjong, Amanda M. Frake, Panuwan Chantawannakul

**Affiliations:** 1 Bee Protection Laboratory, Department of Biology, Faculty of Science, Chiang Mai University, Chiang Mai, 50200 Thailand; 2 Graduate School, Chiang Mai University, Chiang Mai, 50200 Thailand; 3 USDA-ARS, Honey Bee Breeding, Genetics and Physiology Laboratory, Baton Rouge, Louisiana 70820 USA; 4 School of Science, Mae Fah Luang University, Chiang Rai, 57100 Thailand; 5 Environmental Science Research Center, Faculty of Science, Chiang Mai University, Chiang Mai, 50200 Thailand

**Keywords:** Entomology, Animal behaviour

## Abstract

*Tropilaelaps mercedesae* parasitism can cause *Apis mellifera* colony mortality in Asia. Here, we report for the first time that tropilaelaps mites feed on both pre- and post-capped stages of honey bees. Feeding on pre-capped brood may extend their survival outside capped brood cells, especially in areas where brood production is year-round. In this study, we examined the types of injury inflicted by tropilaelaps mites on different stages of honey bees, the survival of adult honey bees, and level of honey bee viruses in 4^th^ instar larvae and prepupae. The injuries inflicted on different developing honey bee stages were visualised by staining with trypan blue. Among pre-capped stages, 4^th^ instar larvae sustained the highest number of wounds (4.6 ± 0.5/larva) while 2^nd^-3^rd^ larval instars had at least two wounds. Consequently, wounds were evident on uninfested capped brood (5^th^-6^th^ instar larvae = 3.91 ± 0.64 wounds; prepupae = 5.25 ± 0.73 wounds). Tropilaelaps mite infestations resulted in 3.4- and 6-fold increases in the number of wounds in 5^th^-6^th^ instar larvae and prepupae as compared to uninfested capped brood, respectively. When wound-inflicted prepupae metamorphosed to white-eyed pupae, all wound scars disappeared with the exuviae. This healing of wounds contributed to the reduction of the number of wounds (≤10) observed on the different pupal stages. Transmission of mite-borne virus such as Deformed Wing Virus (DWV) was also enhanced by mites feeding on early larval stages. DWV and Black Queen Cell Virus (BQCV) were detected in all 4^th^ instar larvae and prepupae analysed. However, viral levels were more pronounced in scarred 4^th^ instar larvae and infested prepupae. The remarkably high numbers of wounds and viral load on scarred or infested developing honey bees may have caused significant weight loss and extensive injuries observed on the abdomen, wings, legs, proboscis and antennae of adult honey bees. Together, the survival of infested honey bees was significantly compromised. This study demonstrates the ability of tropilaelaps mites to inflict profound damage on *A. mellifera* hosts. Effective management approaches need to be developed to mitigate tropilaelaps mite problems.

## Introduction

*Tropilaelaps* spp. are ectoparasites of giant Asian honey bees (*Apis breviligula*, *Apis dorsata* and *Apis laboriosa*), the original hosts of tropilaelaps mites^1,2^. Among the four known species of *Tropilaelaps*, *Tropilaelaps mercedesae* (hereto referred to as tropilaelaps mites) has the widest distribution and is regarded as the most serious parasitic mite of *Apis mellifera*, more so than varroa mites, in much of Asia^2,3^. Higher prevalence of tropilaelaps mites than varroa mites is due to interspecific competition^4^. Recently, we established factors that are contributory to the rapid build up of tropilaelaps mites. After eclosion, unmated females have the ability to produce both male and female offspring, which is probably due to deuterotokous parthenogenesis or may be caused by endosymbiotic bacteria^5^. Hence, the production of females only, which is thought to increase the pool of non-reproductive tropilaelaps mites due to mating failure^6^, may not have a significant negative impact on mite population growth. The symbiotic bacterium, *Rickettsiella grylli*-like, and a part of the *Wolbachia* gene have been detected in tropilaelaps mites in China^7^. *Wolbachia* is the most common reproductive parasite of mites^8^^.^ However, its association with tropilaelaps mites in Thailand has not been established. In addition, both young daughters and foundress mites that were collected from tan-bodied pupae reproduced successfully without undergoing a phoretic period, which can lead to more generations per year^5^.

Varroa mites require hemolymph or fat body intake from their hosts to stimulate reproduction, complete their development, and survive^9^. As a consequence, parasitism of tropilaelaps mites reduces weight at emergence and longevity of adult honey bees under laboratory conditions^10^. Longevity of tropilaelaps mite-infested honey bees in field colonies has not been studied. Mite-feeding also reduces total protein concentration of infested pupae^11^, which may contribute to the alteration of immune responses^12^. Further, feeding by tropilaelaps mites support the proliferation of Deformed Wing Virus (DWV) in the colonies^10,12–14^. However, the major impact of tropilaelaps mite parasitism is probably caused by the mite itself^12^. This was supported by the detection of DWV in DWV-positive pupae infested with DWV-negative mites, and in tropilaelaps mites that are infesting DWV-negative pupae. Hence, some honey bees infected with DWV are asymptomatic or have normal wings^12^. Whether the clinical symptoms that are thought to be associated with DWV are mere manifestations of the degree of feeding injuries sustained by host honey bees or the synergistic effects of both wounds and pathogen load warrants further study. Other viruses such as Black Queen Cell Virus (BQCV) have also been detected in tropilaelaps mite-infested honey bees, but not in tropilaelaps mites^12^. Recently, Acute Bee Paralysis Virus (ABPV) has been detected in *T. mercedesae and A. cerana* in Thailand^15^. While varroa mites create one or two large wounds for communal feeding^16,17^ and possible entry points for these pathogens^18,19^, the types and degree of injuries caused by tropilaelaps mite parasitism have not been investigated.

In the laboratory, *T. clareae* (most likely referring to *T. mercedesae*) are short-lived (~2 days) on adult honey bees^20,21^, but lived longer (~four weeks) when fed unlimited supply of 4^th^ instar larvae^22^. Further, these mites are normally seen scurrying on combs with varying ages of brood especially in highly infested colonies^23^. The relationship between this active movement on combs and prey hunting for increased mite survival when outside brood cell has not been investigated. Therefore, this study was conducted to determine the extent of damages caused by tropilaelaps mites on *A. mellifera*. By examining pre- and post-capped stages of brood from field colonies, we specifically (1) determined if tropilaelaps mites feed on unsealed brood as a result of their high mobility on combs, (2) described the types of injuries sustained by host honey bees (unsealed larvae to adults), and (3) assessed if the incidence of viruses was associated with the presence of wound scars in pre-capped brood, and in uninfested and mite-infested capped brood.

## Results

### Number of wounds on tropilaelaps-infested and uninfested honey bees

**Figure 1:**
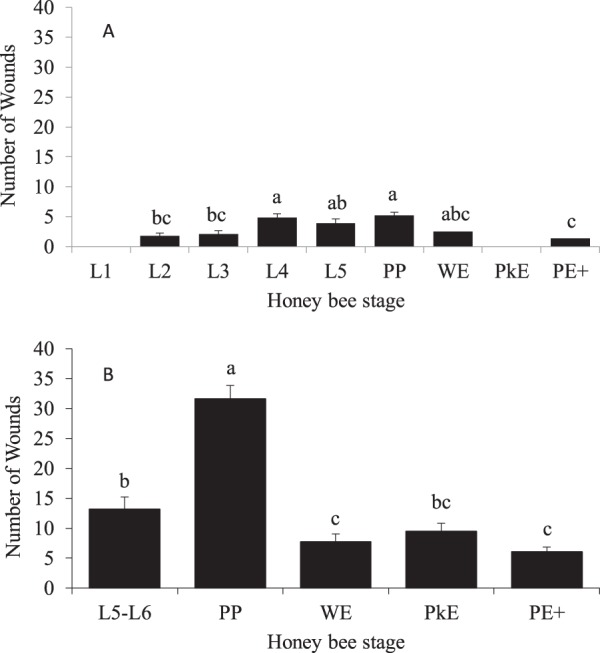
Number of wounds (mean ± SE) observed in different stages of *Apis mellifera*: (**A**) pre-capped and uninfested post-capped stages. (**B**) infested post-capped stages. Bars with the same letters are not significantly different (*P* > 0.05). There were no injuries found in uninfested 1^st^ instar larvae (L1) or pink eyed (PkE) pupae. L1 = 1^st^ instar larva, L2 = 2^nd^ instar larva, L3 = 3^rd^ instar larva, L4 = 4^th^ instar larva, L5 = 5^th^ instar larva, L6 = 6^th^ instar larva, PP = prepupa, WE = white-eyed pupa, PkE = pink-eyed pupa, PE^+^  = purple-eyed and older pupae.

**Figure 2:**
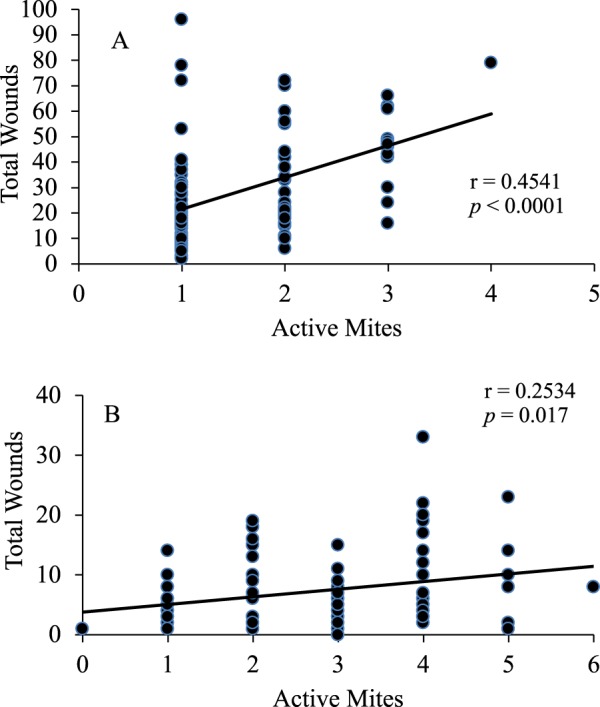
The number of wounds was correlated with the number of actively feeding mites in: (**A**) 5^th^ instar larvae to pre-pupae, and (**B**) white-eyed and older pupae hosts.

**Figure 3:**
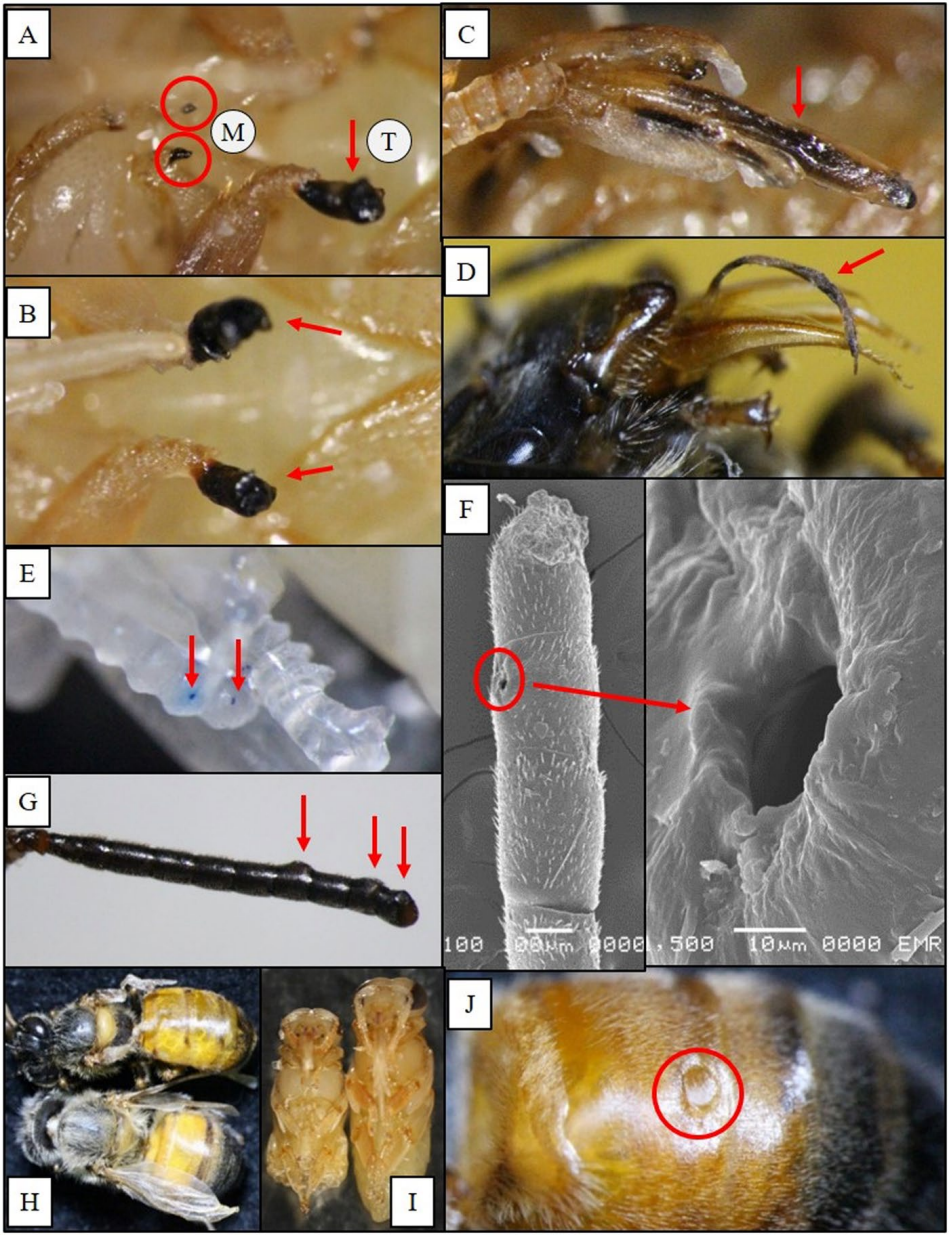
Injuries caused by *Tropilaelaps mercedesae* on pupae and adults. (**A**) Necrotic protuberances of the mesothoracic legs (M) and tarsus (T) of a pupa; (**B**) Necrotic tarsi (claws) of a pupa; (**C**) Darkening of the mouthparts of a pupa; (**D**) Dried proboscis of an adult worker; (**E**) Fresh injuries (stained blue) on antenna of a pupa; (**F**) Scanning electron microscope (SEM) photo of an injury on the antenna (left) of an adult worker and enlarged SEM photo (right); (**G**) Three injured (swollen) flagella of an adult worker; (**H**) two workers with deformed wing; (**I**) retarded growth of an infested pupae (left) and normal growth of uninfested pupa (right); and (**J**) a hole on the tergite of the abdomen. Digital photos by L. I. de Guzman. SEM photos by P. Phokasem.

A total of 3,515 honey bees (426 uncapped brood, 2,520 capped brood and 569 adult workers) were examined for numbers of wounds using a trypan blue staining method. Of the 426 uncapped brood (1^st^-4^th^ instar larvae) examined, 33% sustained injuries. More than half of the 4^th^ instar larvae received 4.9 ± 0.5 wounds per injured honey bee while the 2^nd^-3^rd^ instar larvae supported fewer wounds (about 2 wounds per injured honey bee) (F = 6.25; *P* = 0.0025) (Fig. 1A). No injuries were observed on 1^st^ instar larvae. Feeding injuries were also observed in both the uninfested (n = 2,326) and mite-infested (n = 194) capped brood. However, only 5% of the uninfested capped brood were injured; ~14% of the uninfested prepupae and 7% of uninfested 5^th^-6^th^ instar larvae suffered 5.3 ± 0.7 and 3.9 ± 0.6 wounds, respectively (F = 6.39; *P* = 0.0005) (Fig. 1A). Further, about 90% of these injuries were fresh. Only two uninfested white-eyed pupae had injuries, and none of the 318 uninfested pink-eyed pupae were injured. Of the 194 sealed brood that were infested with tropilaelaps mites, all but one pupa sustained injuries. Infested prepupae showed the most injuries (31.6 ± 2.3 wounds) followed by 5^th^–6^th^ instar larvae (13.2 ± 2.0 wounds) and pink-eyed pupae (9.5 ± 1.3 wounds) (F = 40.70; *P* < 0.0001) (Fig. 1B). White-eyed, and purple-eyed and older pupae suffered ≤8 wounds. Pearson’s correlation analyses showed that the total number of wounds on the 5^th^ instar larvae to prepupae were associated with the number of actively feeding mites (adult and nymph feeding stages) (r = 0.454; *P* < 0.0001) (Fig. 2A). The numbers of wounds on white-eyed and older pupae were also influenced by the number of mites feeding on the honey bees (r = 0.253; *P* < 0.018) (Fig. 2B). High numbers of mites caused wing deformities (Fig. 3H) and stunted growth in infested pupae (Fig. 3I).

### Tropilaelaps feeding location on *A*. *mellifera* host

#### 2^nd^ to 4^th^ instar larvae

**Figure 4:**
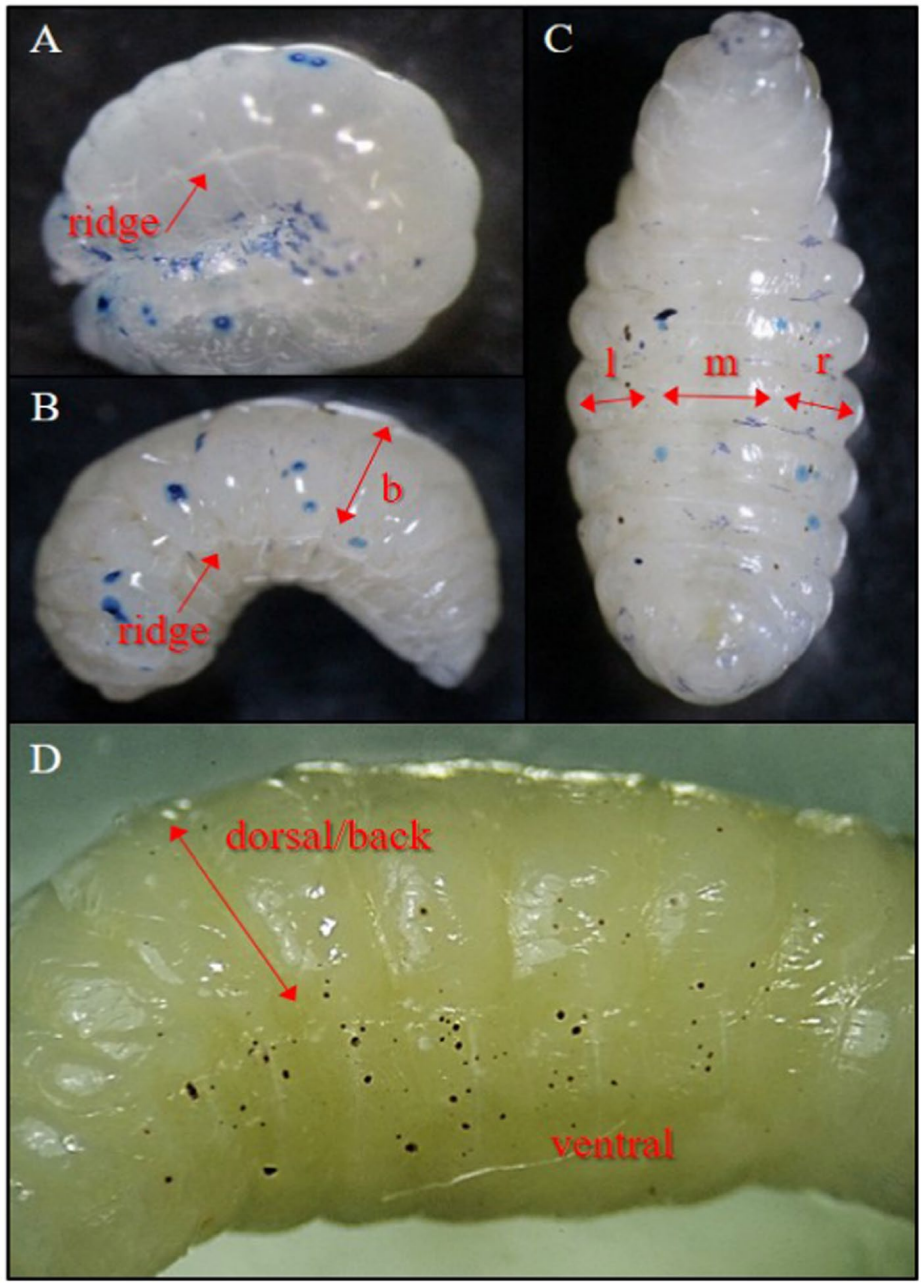
Injuries caused by *Tropilaelaps mercedesae* on larvae and prepupae of *Apis mellifera*. (**A**) Fresh injuries (stained blue) on 2^nd^ instar larva; (**B**) fresh injuries on 4^th^ instar larva; (**C**) fresh and scarred wounds on prepupa, and also shows the left (l), middle (m) and right (r) ventral sections; (**D**) scarred wounds on prepupa infested with tropilaelaps mites and also shows the ventral and dorsal/back (b) of a prepupa. Photos by L. I. de Guzman.

**Figure 5:**
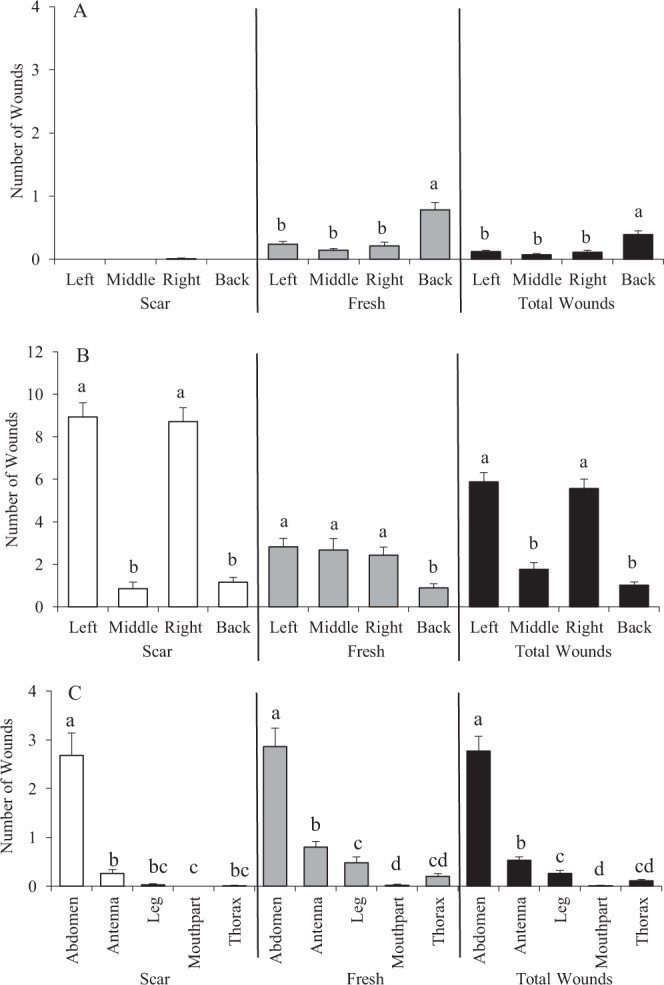
Number (mean ± SE) and location of fresh and scarred wounds on *Apis mellifera* hosts: (**A**) pre-capped stages (2^nd^ to 4^th^ instar larvae); (**B**) infested post-capped stages (5^th^ instar larvae to pre-pupae); and (**C**) infested post-capped stages (white-eyed and older pupae). For each group, bars with the same letters are not significantly different (*P* > 0.05).

The feeding location was facilitated by the position and movement of the developing honey bees, and the activeness of both nymphal and adult stages of tropilaelaps mites within the capped brood cells. On pre-capped stages (2^nd^ -4^th^ instar larvae), wounds were observed on the exposed side (mostly back of the ridges) of the grub-like larvae as they assumed the C position in a pool of royal jelly (Figs 4A,B and 5A).

#### 5^th^ instar larvae to prepupae

After sealing, the larvae moved around the cells and briefly exposed their back to mite feeding. Thereafter, the larvae spun cocoon and developed into prepupae that assumed an elongated position. This elongation (with a slight arch) provided more free spaces for the mites to easily feed on the ventral side of prepupae (Fig. 4C,D). With the presence of protonymphs at this time, the number of injuries (both fresh and scars) increased especially on the left and right sides of prepupae (Figs 4C and 5B). Prior to moulting, most wounds had healed appearing as brown to black scars of various sizes (Fig. 4D).

#### White-eyed and older pupae

White-eyed and older pupae also sustained multiple injuries, especially on their abdomen (Fig. 5C). We also found injuries of the proboscis, a composite organ for drawing liquid such as nectar and water on adult honey bees (Fig. 3C,D). It was common to find tropilaelaps mites (nymphs and adults) on the topmost part of capped brood cells. These mites were probably feeding on the head region of the pupae during this time. The antennae also sustained significant injuries (Fig. 3E–G). On the leg, an important organ for locomotion and also for pollen collection and cleaning of antennae, injuries on the different segments were observed. The basitarsus (pretarsus) and protuberances at the bases of the mesothoracic legs supported most of the injuries (Fig. 3A,B). Sometimes, nymphal stages of tropilaelaps mites were observed between the two mesothoracic legs. Necrotic tissues of the basitarsi of the mesothoracic legs (and sometimes the tip of the antennae) often resulted in amputation as the dead tissues fell off.

#### Adult honey bees

The effects of indiscriminate feeding on pre-capped and post-capped stages were evident on adult honey bees. Of the 329 symptomatic honey bees (with wing deformities or with normal wings but small and sluggish) examined, 39.8% had injured antennae (Fig. 3G), 11.2% with wing deformities, 8.8% suffered abdominal injuries (Fig. 3J), 1.2% had damaged proboscis (Fig. 3D), only 0.6% had injuries on the legs, and none had damaged thorax.

### Weight and survival of adult honey bees

Honey bees that were not deliberately infested with tropilaelaps mites (control) as newly sealed brood were significantly heavier (108.04 ± 2.89 mg, n = 510) than those infested with tropilaelaps mites (93.58 ± 0.37 mg, n = 497) (Mann-Whitney *U* = 41376, *P* ≤ 0.0001). In the infested group, 35.8% had deformed wings and weighed an average of 89.92 ± 0.76 mg (n = 178), which were significantly lighter than those with normal wings (95.10 ± 0.48 mg, n = 319) (Mann-Whitney *U* = 18768, *P* ≤ 0.0001). In addition, we also found 3% of deformed wings in control group. The five-day survival of 596 uninfested and 220 mite-infested honey bees (only those with normal wings) was also monitored. Overall, the survival of honey bees that were deliberately infested with tropilaelaps mites during their capped stages was significantly lower than uninfested honey bees (χ^2^ = 60.77, *P* < 0.0001). Only 17% of the infested honey bees survived for five days as compared to 47% for the uninfested honey bees (control). Moderate survival of uninfested honey bees was probably influenced by mite-feeding during their pre-capped stages.

### Level of honey bee viruses in 4^th^ instar larvae and prepupae

**Figure 6:**
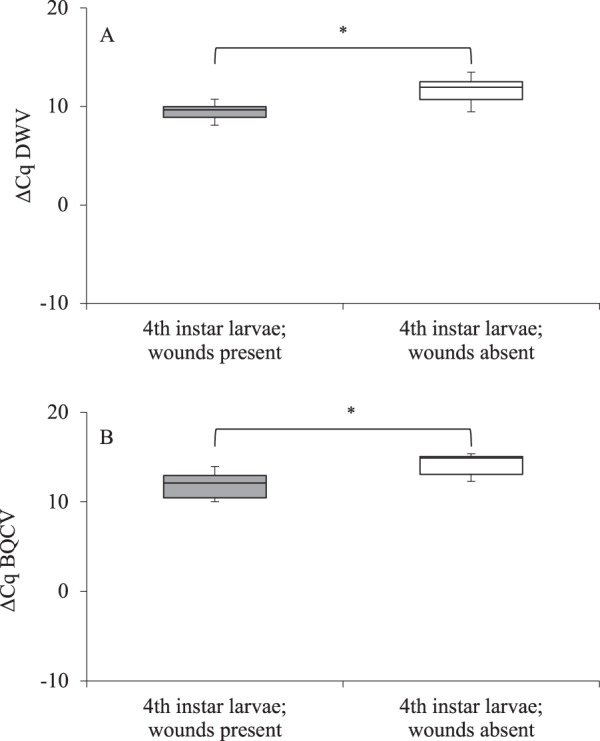
Each box plot represents the ΔCq values for (**A**) Deformed Wings Virus and (**B**) Black Queen Cell Virus in 4^th^ larval instars of *Apis mellifera*. *Significantly different from each other. Cq: qPCR signal for pathogen loads. Lower Cq values mean higher copy numbers of the pathogen.

**Figure 7:**
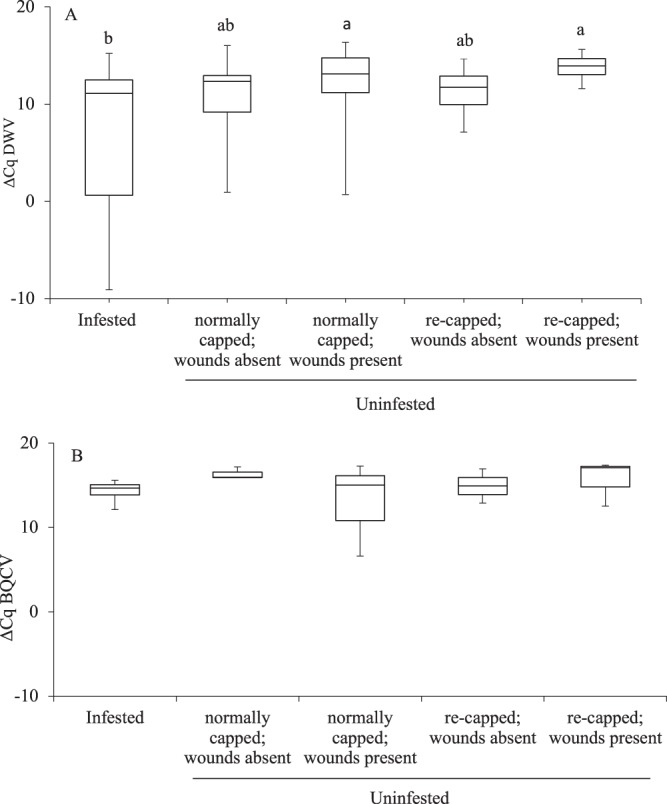
Each box plot represents the ΔCq values for (**A**) Deformed Wings Virus and (**B**) Black Queen Cell Virus in prepupae of *Apis mellifera*. Bars with different letters are significantly different from each other; no letters indicate no significant differences. Cq: qPCR signal for pathogen loads. Lower Cq values mean higher copy numbers of the pathogen.

Quantitative viral analyses of 4^th^ instar larvae and prepupae showed that all samples were negative for Acute Bee Paralysis Virus (ABPV), Chronic Bee Paralysis Virus (CBPV), Kashmir Bee Virus (KBV), Sacbrood Virus (SBV) and Israeli Acute Paralysis Virus (IAPV). However, both Deformed Wing Virus (DWV) and Black Queen Cell Virus (BQCV) were detected. Among the 4^th^ instar larvae, those with wound scars had higher DWV levels than the ones without wound scars (*t* = 3.314; *P* < 0.006) (Fig. 6A). The same trend was observed for BQCV in 4^th^ instar larvae (*t* = 2.329; *P* < 0.048) (Fig. 6B). In the prepupae, DWV levels were highest in the infested group (F = 3.87; *P* < 0.007) (Fig. 7A). The wide variation in the DWV levels of the infested prepupae may indicate that some of them had incurred both pre-and post-capped injuries. The levels of BQCV was not significantly different among treatment groups (F = 0.63; *P* = 0.649) (Fig. 7B).

## Discussion

This study demonstrates that *T. mercedesae* feeds not only on post-capped brood, but also on pre-capped stages of *A. mellifera*. Our results contrast sharply with varroa mites that only start feeding after the sealed larvae consumed their larval food^24^. This feeding on unsealed brood by tropilaelaps mites is facilitated by their high mobility on combs allowing them to quickly find hosts for nourishment and eventually invasion of suitable hosts for reproduction. Results from various cage experiments showed that *T. clareae* (likely referring to *T. mercedesae*) are short-lived (~2 days) on adult honey bees, which supported the interpretation that phoretic mites either enter brood cells immediately or die due to their inability to feed on adult honey bees^20,21,25^. Whether or not tropilaelaps mites are truly phoretic on adult honey bees has not been studied although they appeared to be feeding on soft membranes of the wing axillaries^26^. In general, phoresy on adult honey bees is important in transporting mites to a new host or location or to meet the maturation process of spermatozoa as observed in varroa mites^27^. For tropilaelaps mites, phoresy is not required for successful reproduction^5^. In tropical Asia, continuous brood supply supports uninterrupted mite population growth^28,29^. However, in Asian countries where winter can be harsh, brood production is limited. Nonetheless, tropilaelaps mite infestations persist in cold regions of northern China or South Korea^29,30^, probably due to the mites’ ability to feed on small patches of unsealed brood sufficient enough to extend their longevity until more brood is available. Extended broodless period also happens during the seasonal migration of giant honey bees, the original hosts of tropilaelaps mites. However, the ability of adult tropilaelaps mites to survive extended broodless periods remain unclear. Therefore, the mites’ ability to feed on unsealed brood was previously reported to explain the presence of tropilaelaps mites in newly settled swarm of giant honey bees in Borneo^31^. In addition, honey bee fat body tissue was reported integral to varroa mites diet when feeding^9^. It is possible that earlier feeding on larvae could also benefit tropilaelaps mites for their reproduction, however, no evidence is documented, and further studies are needed to confirm this.

Tropilaelaps mites have chelicerae with a bidentate fixed digit and a spine-like terminal hook on the movable digit^32^. This pair of grasping and tearing organs enables tropilaelaps mites to inflict multiple wounds in both pre- and post-capped brood stages. In contrast, the saw-like blade chelicerae of varroa mites^32^ only makes 1–3 incisions on the integuments of prepupae, and one abdominal wound on pupae with no found injury on unsealed brood^16^. In this study, most injuries caused by tropilaelaps mites were observed on 4^th^ instar larvae (~5 wounds) among the pre-capped stages, and on both mite-infested (~32 wounds) and mite-free (~5 wounds) prepupae among capped brood stages. The presence of wounds on uninfested prepupae confirmed such feeding on unsealed brood, which may amplify the effects of tropilaelaps mite parasitism on honey bee hosts that were fed upon during pre-capped stages and then parasitized during their post-capped stages. Although most wounds on prepupae had healed when they moulted into white-eyed pupae, the combined effect of pre-capped and post-capped brood injuries may have caused the profound damages on adult honey bees that resulted in reduced weight and increased honey bee mortality. In the laboratory, about 40% of mite-infested and >70% of uninfested honey bees were still alive after five days^10^. We observed significantly lower percentage of infested (17%) and uninfested (40%) honey bees that survived after five days under field condition. This low survival of infested honey bees and moderate survival of uninfested honey bees suggests that some of our test honey bees had suffered feeding injuries during their pre-capped stages. The colony sources for our test brood frames had 6–7% brood infestation, which is significantly higher than the colonies (0.29%) used by Khongphinitbunjong *et al*.^10^. This discrepancy in honey bee survival may also be due to the ability of honey bees to expel injured live honey bees in field colonies, an activity that cannot be performed in enclosed laboratory cages. It is common for an injured honey bee to sustain multiple wounds, which may accelerate mortality or removal of injured honey bees from the colonies by house honey bees. Perhaps injured or unhealthy honey bees were quickly expelled from these hygienic colonies or they leaved the hives to reduce rates of disease transmission to nest mates^33^. It is also possible that some of our marked honey bees may have drifted to other colonies. Nevertheless, we did not observe any marked honey bees in any of the other colonies in the apiary.

Honey bees have high-level cognitive abilities and social behaviors which allow them to solve tasks. Our findings suggest, however, that the ability of injured honey bees to perform in- and out-hive tasks such as foraging, taking care of brood, and protecting hives from parasites and predators may be impaired due to their significant injuries caused by tropilaelaps mites. One to three wounds in one or both antennae, deformation or desiccation of the glossa or labium of the proboscis, and one or two holes (mean = 0.11 ± 0.04 mm^2^) on the tergite or pleurite of the abdomen of symptomatic honey bees were observed in this study. Gustatory sensilla are mostly located on these main appendages, which are crucial for honey bees’ survival^34,35^. Malformations to sensilla of the antennae have been observed in workers and drones infested with varroa mites^36^. Like varroa mites, tropilaelaps mites prefer drone brood over worker brood^23,37,38^. Hence, the same injuries can be expected on tropilaelaps mite-infested drones.

Feeding by parasitic mites reduces total protein concentration of infested honey bees^11^, which may contribute to the alteration of immune responses^12^. Hence, transmission and activation of mite-borne viruses such as DWV are facilitated^39–41^. The opened wounds also serve as routes for microbial infection^18^. In this study, the naturally infested honey bees had higher DWV levels than uninfested honey bees, which has also been observed in deliberately infested honey bees^10^. These results support previous reports that tropilaelaps mites are vectors for DWV and promote transmission in honey bees^13,42^, similar to *V. destructor*^43,44^. DWV replication has been reported to occur in tropilaelaps mites^14^ in which females, males and nymphs have equally high DWV levels^7^. Also, high proportions of our stained samples (94–98%) had fresh wounds. Thus, it is likely that the 4^th^ instar larvae and prepupae which were infested by tropilaelaps mites used for viral analyses also had fresh wounds acquired during their pre-capped stages. Hence, the wide variation in the levels of DWV in infested prepupae may suggest that majority of the honey bees were repeated hosts (honey bee brood could be fed during on pre-capped and post capped brood stage, therefore the individual might serve as repeated host), increasing the number of wounds and viral loads. Thus, the additive negative effects of wounds and pathogens may significantly impact honey bees’ ability to survive longer or perform in-and out-hive tasks. It is interesting to note that BQCV levels at early stage honey bee larvae were different depending on numbers of tropilaelaps-wound scars. According to previous reports, tropilaelaps mites only vectored DWV^12,14^, the puncture wounds providing entry to honey bee pathogens and the alteration of honey bees’ immune responses caused by mites may result in different BQCV levels^12^.

Feeding of varroa mites on only larvae within 18 h (worker) and 32 h (drone), respectively, after cell capping activates oogenesis^45^. It is not required for reproduction in tropilaelaps mites^5^. However, it is possible that feeding on unsealed larvae by tropilaelaps mites may also increase their reproductive potential, which requires further investigation. Reproduction in varroa mites is also influenced by hygienic behavior by increasing the proportion of non-reproductive varroa mites^46,47^. However, this activity may not be as effective against tropilaelaps mites as originally suggested^6^. On the other hand, hygienic activities expose both brood and tropilaelaps mites. The opening and re-capping of brood cells by honey bees may allow mites to feed repeatedly on opened brood (or before opened brood is re-capped by honey bees), which may promote viral transmission or infection. This was indicated by the presence of DWV in re-capped, uninfested brood that showed evidences of mite-feeding. Further, some wounds were probably obtained during their pre-capped stages. Combined with the ability of virgin females (that may be exposed from opened or removed purple-eyed or older pupae) to lay males and females without undergoing phoretic phase, tropilaelaps mites population can quickly develop at damaging levels^26^. Managed colonies around the world continue to suffer from a myriad of factors^48,49^. Hence, the accidental introduction of tropilaelaps mites outside their current range will have an immense impact on worlwide agriculture. Yet, information on all aspects of tropilaelaps mites’ biology and ecology is incomplete^26^. These findings are useful for honey bee researchers attempting to understand tropilaelaps mites’ biology, which may lead to the development of effective ways of managing them. Improved honey bee health and survival also play an important role in agricultural and food production as well as maintaining ecosystem especially in South East Asia where the honey bee parasitic mites are present.

## Materials and Methods

This study was conducted in Chiang Mai University, Thailand between February 2017 and March 2018 using *A. mellifera* colonies.

### Source of honey bees

Eight queen-right Langstroth hive size colonies consisting of 8–10 deep frames were used in this study. These colonies were naturally infested with *T. mercedesae* having brood infestation rates ranging from 6 to 46%, which were estimated following standard protocol^50^. In these colonies, free-roaming mites were observed especially on frames with opened brood. For each colony, brood frames containing 1^st^ to 6^th^ instar larvae, prepupae, white-eyed pupae, pink-eyed pupae, purple-eyed pupae and older pupae were examined under a dissecting microscope. The youngest larval stages (1^st^-3^rd^ instar larvae) were collected from the cells using a spatula while older brood was removed from each cell by destroying the cell walls around and lifting the brood with blunt-tipped forceps. Before opening each capped brood cell, the wax capping was examined for recapping; recapped brood has elevated dark brown wax capping and no cocoon^51^. If a capped brood was infested, the number and stages of mites were noted. From each colony, symptomatic honey bees (with wing deformities or with normal wings but small and sluggish) were hand-picked from brood frames before a scoop of random honey bees (~50) were collected for examination (Supplementary Information Table S1).

### Observations on the number and location of injuries

Each of the pre- and post-capped honey bees was immersed in 0.4% trypan blue solution (Invitrogen™) for 30 min at room temperature. This procedure stained epidermal cells that were damaged by tropilaelaps mites^16^. Fresh integumental wounds appeared as blue spots and wounds that had healed were brown to black in color (referred to here as wound scars). To determine the location of injuries on 1^st^ instar larvae to prepupae, each honey bee was divided into ventral (left, middle and right) and dorsal (back) (Fig. 4A–D), and the specific injured body parts on pupae were identified. Examination was conducted under a stereo microscope. Photographs were taken using a digital camera attached to the microscope, and scanning electron microscope using standard protocols^52^.

### Weight and survival of adult honey bees

Out of the eight colonies used in section a, frames containing newly sealed brood from three colonies with the lowest infestations (6–7% brood infestations, Supplementary Information Table S1) were inoculated with tropilaelaps mites using the transfer technique (a mite was inserted to newly sealed brood via a small incision (~1 mm) which made from an insect pin and then close opening using an insect brush)^6,47,53^. A mixture of free-roaming and brood mites was used for brood inoculation. For each colony, newly sealed brood (n = 200) was deliberately inoculated with one foundress; non-inoculated brood (n = 200) served as control. Our method followed Khongphinitbunjong *et al*.^10^, which showed no effect of artificial manipulation on weight and longevity of honey bees in cage experiments. Thereafter, test frames were kept in an incubator (34 °C, 60% relative humidity). Ten days post inoculation, each brood was opened to count the number of mite progeny. Each pupa was placed in a ventilated microcentrifuge tube for emergence in the incubator^54^. Upon emergence, the individual honey bee was weighed. To monitor survival, only those honey bees with normal wings were marked on the thorax with enamel paint, introduced into a host colony using a screen cage to facilitate acceptance, and then released the following day. On the 5^th^ day, honey bee survival was determined by conducting a census of all honey bees in the host colony. Monitoring halted at five days because only few infested honey bees survived at that time.

### Viral analyses

Honey bees for the viral analyses were collected from one of the eight colonies that had 18% brood infestation (Supplementary Information Table S1). For the pre-capped stages, 15 4^th^ instar larvae (seven with wound scars and eight without wound scars) were collected whereas naturally infested (n = 30) and uninfested (n = 120) prepupae represented the post-capped stages. Uninfested prepupae were further categorized as: (a) normally capped with wound scars (n = 30), (b) normally capped without wound scars (n = 30), (c) re-capped with wound scars (n = 30), and (d) re-capped without wound scars (n = 30). Prepupae were selected for the post-capped stage because this stage sustained the most injuries. Also, most injuries had healed as brown to black scars, which facilitated counting of wounds. Wound scars disappeared with shed skin during ecdysis of prepupae to white-eyed pupae. Nevertheless, fresh wounds were likely present in both stages, which can only be verified by staining honey bees. Majority (94–98%) of our stained 4^th^ instar larvae and prepupae samples sustained fresh wounds. All samples for the viral analyses were not stained to prevent contamination.

Total RNA was extracted from individual honey bees using the TRIzol reagent (Invitrogen, Carlsbad, CA, USA) according to the manufacturer’s instructions. RNA was stored in RNase-free water at −80 °C prior to reverse transcription. Reverse transcription reactions for cDNA synthesis were performed using a Tero cDNA Synthesis Kit (Bioline). The presence of seven honey bee viruses: Acute Bee Paralysis Virus (ABPV), Black Queen Cell Virus (BQCV), Chronic Bee Paralysis Virus (CBPV), Deformed Wing Virus (DWV), Israeli Acute Paralysis Virus (IAPV), Kashmir Bee Virus (KBV), and Sacbrood Virus (SBV) (Supplementary Information Table S2) was determined by qPCR using IQ5 Real-Time PCR thermal cycler (Bio-Rad). Amplification was performed in 20 μl reaction volumes using SensiFAS™ SYBR^®^ No-ROX kit (Bioline), consisting of 10 μl 2x SensiFAS™ SYBR^®^ No-ROX mix, 0.8 μl of 10 μM of each primer and *β*-actin^55^ (Supplementary Information Table S2), 7.4 μl of nuclease free water and 1 μl cDNA. Reactions were run at 95 °C for 5 min, 40 cycles of 95 °C for 10 s, 59 °C for 5 s followed by a melt-curve dissociation analysis. All reactions included duplicates. The negative control (no template) was included in each run of the reaction. The qPCR data were expressed as the threshold cycle (Cq) value and were calculated by using the Cq value of the reference gene (*β*-actin) to the target genes (ΔCq)^56–58^.

### Statistical analyses

A one-way analysis of variance (ANOVA) was used to compare the total number of wounds sustained by the different pre-capped and post-capped stages and also to determine preference for feeding. Data on total number of wounds were square-root transformed prior to analyses to better approximate normality. Frequency tables and Fisher’s chi-square were used to analyze proportions of the total number of wounds within number of injured honey bees (PROC FREQ, SAS v9.4). A Pearson’s correlation analysis was performed to test the relationship between the number of active mites and total wounds for the different honey bee stages^59^. The Mann-Whitney *U* test was used to compare emergence weights of infested and uninfested honey bees. Data on virus levels of prepupae were normally distributed and were subjected to a one-way ANOVA to compare differences across treatment groups. Where differences were found, means were compared using a Tukey-HSD with a 95% confidence. DWV and BQCV levels of 4^th^ instar larvae with or without wound scars were compared using a *t*-test^60^.

## Supplementary information

S1 and S2
