# Supplementary material for: Feeding by Tropilaelaps mercedesae on pre- and post-capped brood increases damage to Apis mellifera colonies
Source: Sci Rep. 2019 Sep 10;9:13044. doi: 10.1038/s41598-019-49662-4 (PMC6737106; doi:10.1038/s41598-019-49662-4)
Supplement: Supplementary file 1 — S1 and S2 [file 41598_2019_49662_MOESM1_ESM.docx]

**Supplementary Information**

Feeding by *Tropilaelaps mercedesae* on pre- and post-capped brood increases damage to *Apis mellifera* colonies

Patcharin Phokasem^1,2^, Lilia I. de Guzman^3,*^, Kitiphong Khongphinitbunjong^4^, Amanda M. Frake^3^, Panuwan Chantawannakul^1,5,*^

^1^Bee Protection Laboratory, Department of Biology, Faculty of Science, Chiang Mai University, 50200, Thailand.

^2^Graduate School, Chiang Mai University, Chiang Mai 50200, Thailand.

^3^USDA-ARS, Honey Bee Breeding, Genetics and Physiology Laboratory, Baton Rouge, Louisiana 70820, USA.

^4^School of Science, Mae Fah Luang University, Chiang Rai 57100, Thailand.

^5^Environmental Science Research Center, Faculty of Science, Chiang Mai University, 50200, Thailand.

*Authors for correspondence: Lilia I. de Guzman, e-mail: [lilia.deguzman@usda.gov](mailto:lilia.deguzman@usda.gov) and Panuwan Chantawannakul, e-mail: [panuwan@gmail.com](mailto:panuwan@gmail.com)

**Table S1** Percentage of tropilaelaps infestation, normal capped cells, and recapped cells in *Apis mellifera* colonies corresponded to experimental manipulation

| **Colony no.** | **Experimental manipulation** | **Tropilaelaps infestation (%)** | **Normal capped cells**  **(%)** | **Recapped cells**  **(%)** |
| --- | --- | --- | --- | --- |
| 1 | - Number of wounds on tropilaelaps-infested and uninfested honey bees  - Tropilaelaps feeding location on *Apis mellifera* host | 6 | 94 (n = 275) | 6 (n = 18) |
| 2 | - Number of wounds on tropilaelaps-infested and uninfested honey bees  - Tropilaelaps feeding location on *Apis mellifera* host | 20 | 78 (n = 270) | 22 (n = 78) |
| 3 | - Number of wounds on tropilaelaps-infested and uninfested honey bees  - Tropilaelaps feeding location on *Apis mellifera* host  - Weight and survival of adult honey bees | 6 | 93 (n = 470) | 7 (n = 38) |
| 4 | - Number of wounds on tropilaelaps-infested and uninfested honey bees  - Tropilaelaps feeding location on *Apis mellifera* host | 46 | 41 (n = 294) | 59 (n =384) |
| 5 | - Number of wounds on tropilaelaps-infested and uninfested honey bees  - Tropilaelaps feeding location on *Apis mellifera* host | 27 | 79 (n =352) | 21 (n = 96) |
| 6 | - Number of wounds on tropilaelaps-infested and uninfested honey bees  - Tropilaelaps feeding location on *Apis mellifera* host  - Viral analyse | 18 | 82 (n =295) | 18 (n = 65) |
| 7 | - Number of wounds on tropilaelaps-infested and uninfested honey bees  - Tropilaelaps feeding location on *Apis mellifera* host  - Weight and survival of adult honey bees | 7 | 92 (n = 424) | 8 (n = 39) |
| 8 | - Number of wounds on tropilaelaps-infested and uninfested honey bees  -Tropilaelaps feeding location on *Apis mellifera* host  - Weight and survival of adult honey bees | 6 | 93 (n = 414) | 7 (n = 33) |

**Table S2** Primers used in this study

| **Virus** | **Primers** | **Sequence (5'-3')** | **Size** | **Reference** |
| --- | --- | --- | --- | --- |
| DWV | DWV-F8668 | TTCATTAAAGCCACCTGGAACATC | 136 | 55 |
|  | DWV-B8757 | TTTCCTCATTAACTGTGTCGTTGA |  |  |
| BQCV | BQCV-qF7893 | AGTGGCGGAGATGTATGC | 294 | 55 |
|  | BQCV-qB8150 | GGAGGTGAAGTGGCTATATC |  |  |
| KBV | KBV-F6639 | CCATACCTGCTGATAACC | 200 | 55 |
|  | KIABPV-B6707 | CTGAATAATACTGTGCGTATC |  |  |
| SBV | SBV-qF3164 | GCTCTAACCTCGCATCAAC | 335 | 55 |
|  | SBV-qB3461 | TTGGAACTACGCATTCTCTG |  |  |
| ABPV | ABPV-F6548 | GATACCCCCATGGCTC | 197 | 55 |
|  | KIABPV-B6707 | CTGAATAATACTGTGCGTATC |  |  |
| CBPV | CBPV1-qF1818 | CAA CCTGCCTCA ACACAG | 296 | 55 |
|  | CBPV1-qB2077 | AAT CTGGCAAGG TTGACTGG |  |  |
| IAPV | IAPV-F6627 | CCA TGCCTGGCG ATTCAC | 203 | 55 |
|  | KIABPV-B6707 | CTG AATAATACTGTG CGTATC |  |  |
| *β*-actin | Am-actin2-qF | CGACGGTCAGGTCATCAC | 271 | 55 |
|  | Am-actin2-qB | GTTGAGGGAGCCAAAGAGG |  |  |
